# Understanding the Role of Purinergic P2X7 Receptors in the Gastrointestinal System: A Systematic Review

**DOI:** 10.3389/fphar.2021.786579

**Published:** 2021-12-20

**Authors:** Nathalie Cheng, Li Zhang, Lu Liu

**Affiliations:** ^1^ School of Medical Sciences, UNSW Sydney, Sydney, NSW, Australia; ^2^ School of Biotechnology and Biomolecular Sciences, UNSW Sydney, Sydney, NSW, Australia

**Keywords:** purinergic P2X7 receptor, gastrointestinal system, inflammation, inflammatory bowel disease, immune cell regulation, infection

## Abstract

**Background:** The role of purinergic P2X7 receptor (P2X7R) is of interest due to its involvement in inflammation and mediating immune cell responses. P2X7R is particularly implicated in the development of inflammatory bowel disease (IBD). However, the extent of the actions of P2X7R in the gastrointestinal (GI) system under physiological and pathophysiological conditions remains to be elucidated. This systematic review aimed to identify, summarize and evaluate the evidence for a critical role of P2X7R in the GI system.

**Methods:** We searched PubMed, Embase and Scopus with search terms pertained to P2X7R in the GI system in disease or physiological state, including “P2X7 or P2X7 receptor or purinergic signaling” in combination with any of the terms “intestine or colon or gut or gastrointestinal,” “pathology or inflammation or disease or disorder,” and “physiology or expression.” Titles and abstracts were screened for potentially eligible full texts, and animal and human studies published in English were included in this study. Data were extracted from papers meeting inclusion criteria. Meta-analysis was not feasible given the study diversity.

**Results:** There were 48 papers included in this review. We identified 14 experimental colitis models, three sepsis models and one ischemia-reperfusion injury model. Among them, 11 studies examined P2X7R in GI infections, six studies on immune cell regulation, four studies on GI inflammation, two studies on GI malignancies, three studies involving intestinal injury due to various causes, two studies on ATP-activated P2X7R in the GI system and two studies on metabolic regulation.

**Conclusion:** Evidence supports P2X7R mediating inflammation and immune cell responses in GI inflammation, infections and injury due to IBD and other challenges to the intestinal wall. P2X7R inhibition by gene knockout or by application of P2X7R antagonists can reduce tissue damage by suppressing inflammation. P2X7R is also implicated in GI malignancies and glucose and lipid homeostasis. P2X7R blockade, however, did not always lead to beneficial outcomes in the various pathological models of study.

## Introduction

Extracellular adenosine triphosphate (ATP) is recognized as an important signaling molecule; it is involved in basic physiological and pathophysiological processes such as tissue homeostasis, wound healing, neurodegeneration, immune and inflammatory responses and cancer (Di Virgilio and Adinolfi, 2016). Despite being a ubiquitous molecule, ATP acts as a co-transmitter in the peripheral and central nervous system (Burnstock, 2012) and it is involved in various cellular responses, including upregulating or inhibiting cell death, regulating growth factors and inflammatory mediators (Di Virgilio and Adinolfi, 2016). ATP is released *via* a combination of vesicular exocytosis, connexin and pannexin hemichannels (Lazarowski, 2012) in cell death, cell stress, in response to mechanical changes, hypoxia or other agents (Bodin and Burnstock, 2001; Burnstock, 2014). The concentration of extracellular ATP is maintained at low nM levels under normal physiological conditions (Di Virgilio and Adinolfi, 2016; Morciano et al., 2017), but this can drastically increase to a few hundred μM in cell damage or death (Di Virgilio and Adinolfi, 2016).

Short- and long-term purinergic signaling is involved in physiological and pathophysiological processes. Short-term signaling mediates neuromodulation, neurotransmission, secretion, chemotaxis and platelet aggregation (Burnstock, 2014). Long-term signaling involves regulating gene expression, cell proliferation, differentiation and apoptosis (Burnstock, 2012; 2014).

ATP exerts its actions *via* purinergic P2 receptors. P2 receptors are classified into P2X and P2Y subfamilies. P2X receptors are ligand-gated ion channels, and P2Y receptors are G protein-coupled receptors. They are further classified into seven P2X subtypes and eight P2Y subtypes (Burnstock, 2006; Kolachala et al., 2007). Different cell types usually express multiple purinergic receptor subtypes (Burnstock, 2012).

Among the seven P2X receptors (P2X1-7), the P2X7 receptor (P2X7R) is of particular interest in inflammation, with evidence showing its role in the inflammatory process (Kopp et al., 2019). Extracellular nucleotides, including ATP and their receptors, have been implicated in the pathogenesis of inflammatory bowel disease (IBD) (Burnstock, 2006; Longhi et al., 2017). P2X7R is the only subtype resistant to desensitization–it can be activated for a long duration, resulting in prolonged effects of its activation (North, 2002; Diezmos et al., 2016). P2X7R has a longer C-terminus (240 amino acids) compared to other P2X receptors (29–87 amino acids). This possibly contributes to the physiological and pathophysiological functions of P2X7R, e.g., activation of lipases, kinases, transcription factors, cytokine release, apoptosis and changes in plasma membrane composition and morphology (Kopp et al., 2019), especially under conditions of inflammatory stress and cell damage whereby the increase in extracellular ATP levels lead to P2X7R activation (Longhi et al., 2017).

Apart from IBD, P2X7R has also been implicated in other gastrointestinal (GI) pathologies, especially for its role in aberrant inflammation when activated (Savio et al., 2018). In infections, malignancy, neurodegeneration and neuroinflammation, P2X7R can have differing actions that may have protective effects or contribute towards disease progression (Savio et al., 2018).

The topic of P2X7R in inflammatory diseases has gained growing interest in recent years. To the best of our knowledge, no systematic review concerning this topic has been published. Thus, the aim of this article is to identify, summarize and evaluate the roles and actions of P2X7R in the GI system.

## Methods

### Search Strategy

We searched PubMed, Embase and Scopus using the terms “P2X7 or P2X7 receptor or purinergic signaling” in combination with any of the terms “intestine or colon or gut or gastrointestinal,” “pathology or inflammation or disease or disorder,” and “physiology or expression.” There were no date limits, but only studies published in English were included. Titles and abstracts were screened, and full-text articles were obtained for potentially eligible studies.

### Inclusion Criteria

Eligible studies were animal or human experimental studies undertaken with a focus on the role of P2X7R. Studies were limited to those involving the GI system. The criteria for methodological rigor of experimental articles included the reporting of species, strain, gender and age of animals (for animal studies), tissue and cell line used, clear description of the experimental protocol with inclusion of proper controls, and reporting the type of statistical analysis undertaken.

### Search Results

**FIGURE 1:**
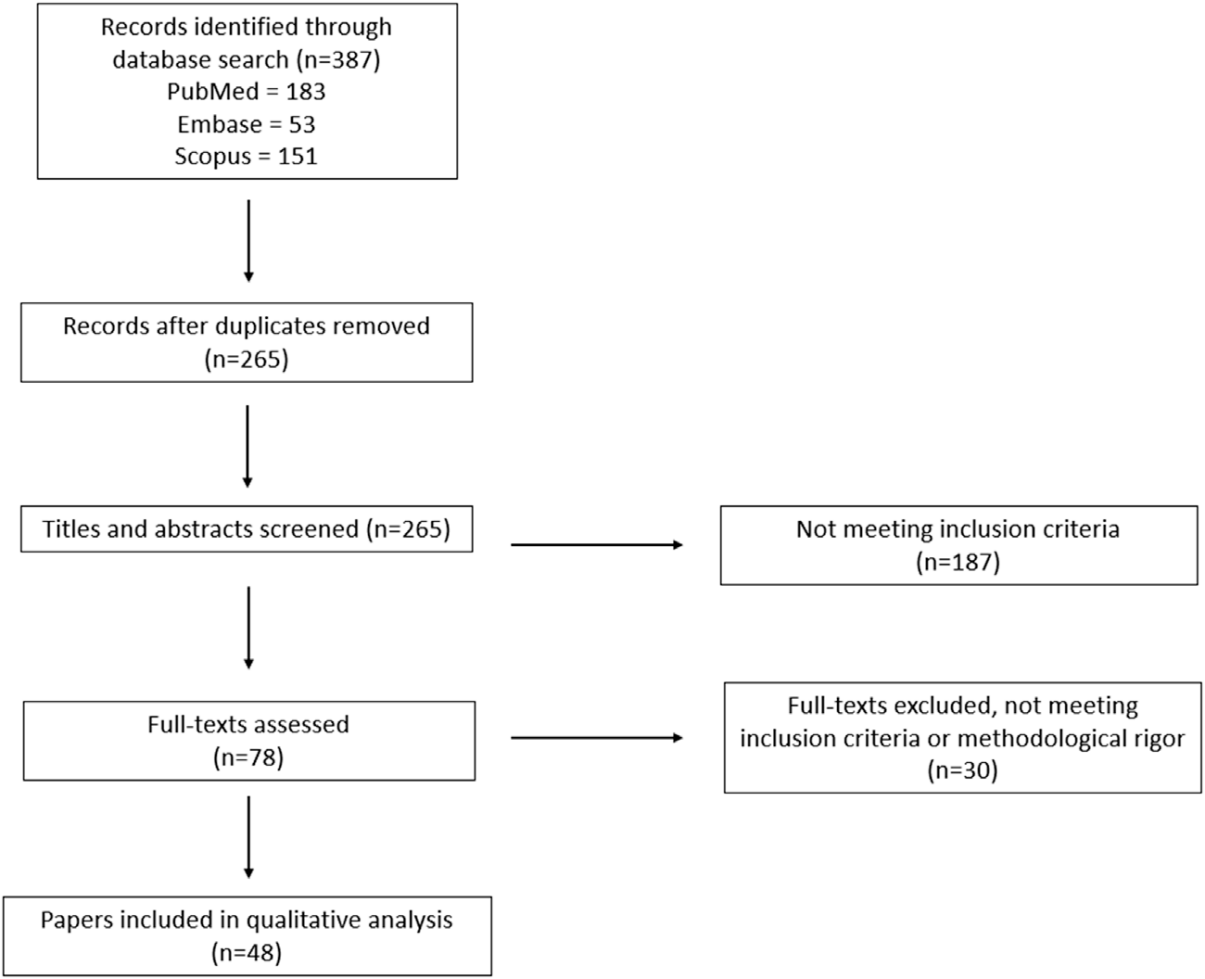
PRISMA flowchart outlining the protocol used in this systematic review.

The search identified a total of 387 records; after eliminating duplicate results, the titles and abstracts of the remaining 265 unique records were screened, of which 78 were selected for full-text review. After a full-text review, 30 did not meet the inclusion criteria and 48 were included in the review. A PRISMA flow chart of the search results is shown in Figure 1.

## Results

There were 48 papers included in this study. We identified 14 experimental colitis models, three sepsis models and one ischemia-reperfusion injury model. Among them, 11 studies examined P2X7R in GI infections, six studies on immune cell regulation, four studies on GI inflammation, two studies on GI malignancies, three studies involving intestinal injury due to various causes, two studies on ATP-activated P2X7R in the GI system and two studies on metabolic regulation.

P2X7R activation has been shown to induce a broad array of cellular responses, including cytokine release, apoptosis, and cell death, which are associated with inflammatory processes. The key actions of P2X7R in the GI system are summarized in Table 1.

**TABLE 1:** Table summarizing action of P2X7R in the GI system.

Action of P2X7R in the GI system		References	Study method
Induces cell death by apoptosis	Intestinal epithelial cells (IECs)	Souza et al. (2012)	*In vitro* studies using human ileocecal adenocarcinoma cell line (HCT8), involving pharmacological inhibition of P2X7R
		Marques et al. (2014)	*In vivo* animal studies; colitis model using 2,4,6-trinitrobenzenesulphonic acid solution (TNBS), involving pharmacological inhibition of P2X7R
		Zhang et al. (2018)	*In vivo* and *in vitro* animal studies (rat intestinal epithelial cell lines IEC-6 and CRL-159) in lipopolysaccharide (LPS) stimulation, involving pharmacological inhibition of P2X7R
		Neves et al. (2014)	*In vivo* animal studies, *in vitro* and *ex vivo* human studies [inflamed mucosal tissues from patients with IBD and macroscopically normal mucosal specimens from the same patients with Crohn’s disease (CD)], colitis model using TNBS or dextran sulfate sodium (DSS), involving P2X7R knockout (KO) mice, pharmacological inhibition of P2X7R and anti-P2X7R antibodies
	Enteric neurons	Gulbransen et al. (2012)	*In vivo* animal studies and *in vitro* human studies (colon tissue from healthy patients and patients with ulcerative colitis (UC), CD and colon cancer); colitis model using 2,4-dinitrobenzenesulphonic acid (DNBS), oxazolone and DSS, involving pharmacological inhibition of P2X7R
		Delvalle et al. (2018)	*In vivo* animal studies; colitis model using DNBS, involving pharmacological inhibition of P2X7R
	Invariant natural killer cells (iNKT)	Liu and Kim (2019)	*In vivo* animal studies; vitamin A and iNKT studies, involving P2X7R KO mice
Controls population of T cells	T regulatory (Treg) cell death	Figliuolo et al. (2017)	*In vivo* and *in vitro* animal studies; colitis model using TNBS or oxazolone, involving P2X7R KO mice and pharmacological inhibition of P2X7R
	Limits expansion of T follicular helper (Tfh) cell population	Proietti et al. (2014)	*In vivo* animal studies and *in vitro* human studies (Peyer’s patches tissues and Tfh-cell-dependent B cell), involving P2X7R KO mice and pharmacological inhibition of P2X7R
		Proietti et al. (2019)	*In vivo* animal studies; using P2X7 KO mice, involving live and attenuated vaccines expressing *Shigella flexneri* periplasmic ATP-diphosphohydrolase (apyrase)
		Perruzza et al. (2017)	*In vivo* animal studies, microbiota transplantation, involving P2X7R KO mice
		Perruzza et al. (2019)	*In vivo* animal studies, involving P2X7R KO mice
	Depletion of tissue-resident memory T cells (T_RM_)	Stark et al. (2018)	*In vivo* animal studies and *in vitro* human and animal studies; experimental infection model and sterile induction of tissue damage using acetaminophen. The study involves inhibition of P2X7R and P2X7R KO mice
	Depletes intestinal Th1 and Th17 CD4^+^ T cells	Hashimoto-Hill et al. (2017)	*In vivo* and *in vitro* animal studies; vitamin A, infection (using *Citrobacter rodentium*), and colitis studies (using CD4^+^ T cells), involving P2X7R KO and Rag1 KO mice
Induces changes in cell morphology		Souza et al. (2012)	*In vitro* studies using human ileocecal adenocarcinoma cell line (HCT8), involving pharmacological inhibition of P2X7R
Induces cell permeability		de Campos et al. (2012)	*In vitro* animal studies; permeability assay involving P2X7R KO mice
Induces reactive oxygen species (ROS) production		Souza et al. (2012)	*In vitro* studies using human ileocecal adenocarcinoma cell line (HCT8), involving pharmacological inhibition of P2X7R
		de Campos et al. (2012)	*In vitro* animal studies; permeability assay involving P2X7R KO mice
Involved in the development of IBD (including colitis models)		Figliuolo et al. (2017)	*In vivo* and *in vitro* animal studies; colitis model using TNBS or oxazolone involving P2X7R KO mice and pharmacological inhibition of P2X7R
		Marques et al. (2014)	*In vivo* animal studies; colitis model using TNBS involving pharmacological inhibition of P2X7R
		Ohbori et al. (2017)	*In vivo* animal studies; colitis model using DSS, involving pharmacological inhibition of P2X7R
		Diezmos et al. (2018)	*Ex vivo* human studies (healthy human colonic muscle strips); colitis model using pro-inflammatory cytokines [tumor necrosis factor (TNF)-α and interleukin (IL)-1β] involving P2X7R antagonist
		Souza et al. (2020)	*In vivo* animal studies; colitis model using TNBS involving pharmacological inhibition of P2X7R
		Gulbransen et al. (2012)	*In vivo* animal studies and *in vitro* human studies (colon tissue from healthy patients and patients with UC, CD and colon cancer); colitis model using DNBS, oxazolone and DSS involving pharmacological inhibition of P2X7R
		Kurashima et al. (2012)	*In vivo* animal studies and *in vitro* human studies (colon tissue from healthy patients and patients with UC and CD); colitis model using TNBS or DSS, involving anti-P2X7R monoclonal antibodies (mAb)
		Neves et al. (2014)	*In vivo* animal studies, *in vitro* and *ex vivo* human studies (inflamed mucosal tissues from patients with IBD and macroscopically normal mucosal specimens from the same patients with CD), colitis model using TNBS or DSS involving P2X7R KO mice, pharmacological inhibition of P2X7R and anti-P2X7R antibodies
		Wan et al. (2016)	*In vivo* animal studies; colitis model using DSS involving pharmacological inhibition of P2X7R
		Hofman et al. (2015)	*In vivo* animal studies; colitis and colitis-associated cancer model using DSS and azoxymethane involving P2X7R KO mice and pharmacological inhibition of P2X7R
Triggers immune cell infiltration	At sites of inflammation	Figliuolo et al. (2017)	*In vivo* and *in vitro* animal studies; colitis model using TNBS or oxazolone involving P2X7R KO mice and pharmacological inhibition of P2X7R
		Marques et al. (2014)	*In vivo* animal studies; colitis model using TNBS involving pharmacological inhibition of P2X7R
		Ohbori et al. (2017)	*In vivo* animal studies; colitis model using DSS, involving pharmacological inhibition of P2X7R
		Su et al. (2019)	*In vivo* animal studies; chronic plus binge alcohol feeding model involving pharmacological inhibition of P2X7R
	At site of infection	Huang et al. (2017)	*In vivo* and *in vitro* animal studies; infection model using *Toxoplasma gondii* and *Trichinella spiralis* involving P2X7R KO mice and pharmacological inhibition of P2X7R
	In ischemia reperfusion injury (ISR)	Palombit et al. (2019)	*In vivo* animal studies; ISR model involving pharmacological inhibition of P2X7R
	In tumor mass	Adinolfi et al. (2015)	*In vivo* and *in vitro* animal studies (B16 melanoma and CT26 colon carcinoma cells); tumor model involving P2X7R KO mice
Induces production of pro-inflammatory cytokines	TNF-α and IL-1β	Marques et al. (2014)	*In vivo* animal studies; colitis model using TNBS involving pharmacological inhibition of P2X7R
		Neves et al. (2014)	*In vivo* animal studies, *in vitro* and *ex vivo* human studies (inflamed mucosal tissues from patients with IBD and macroscopically normal mucosal specimens from the same patients with CD), colitis model using TNBS or DSS involving P2X7R KO mice, pharmacological inhibition of P2X7R and anti-P2X7R antibodies
	TNF and IL-1β	Wan et al. (2016)	*In vivo* animal studies; colitis model using DSS involving pharmacological inhibition of P2X7R
	TNF-α and IL-6	Huang et al. (2017)	*In vivo* and *in vitro* animal studies; infection model using *T. gondii* and *T. spiralis* involving P2X7R KO mice and pharmacological inhibition of P2X7R
		Wu et al. (2017)	*In vivo* and *in vitro* animal studies; sepsis model involving pharmacological inhibition of P2X7R and P2X7R agonist
		Bhave et al. (2017)	*In vivo* and *in vitro* animal studies (rat enteric glial cell line CRL2690) in a prolonged morphine treatment model involving pharmacological inhibition of P2X7R
	TNF-α, IL-1β and IL-6	Su et al. (2019)	*In vivo* animal studies; chronic plus binge alcohol feeding model involving pharmacological inhibition of P2X7R
	IL-1β	de Campos et al. (2012)	*In vitro* animal studies; permeability assay involving P2X7R KO mice
		Cesaro et al. (2010)	*In vitro* human studies (IEC cell line T84, colon specimens from healthy patients and patients with IBD) involving pharmacological inhibition of P2X7R
		Keating et al. (2011)	*In vivo* animal studies on post-infectious visceral hypersensitivity in *T. spiralis* infection involving P2X7R KO mice
	IL-6	Kim et al. (2015)	*In vitro* human studies involving human colon carcinoma cell line (Caco-2) and Burkitt’s lymphoma cell line (Raji) and P2X7R agonist (ATP and LL-37)
	Chemokine ligand 20 (CCL20)	Sim et al. (2018)	*In vitro* human studies using human IEC lines Caco-2 and HT-29 in killed whole-cell oral cholera vaccine (Shanchol^TM^) and short-chain fatty acids (butyrate, acetate and propionate)
	IL-33	Kataoka et al. (2021)	*In vitro* animal studies using mouse dendritic cell line DC2.4 infected with *Citrobacter koseri,* involving pharmacological inhibition of P2X7R
Reduces production of anti-inflammatory cytokines	IL-10 and transforming growth factor (TGF)-β1	Figliuolo et al. (2017)	*In vivo* and *in vitro* animal studies; colitis model using TNBS or oxazolone, involving P2X7R KO mice and pharmacological inhibition of P2X7R
Inhibits TNF-α production		Coquenlorge et al. (2014)	*In vitro* animal (rat enteric nervous system primary culture) and human (longitudinal muscle-myenteric plexus) studies; LPS challenge involving pharmacological inhibition of P2X7R
Activates mast cells	To secrete IL-33	Shimokawa et al. (2017)	*In vivo* and *in vitro* animal studies; infection model using *Heligmosomoides polygyrus* (Hp) involving Spib^-/-^ mice and pharmacological inhibition of P2X7R
	To promote IL-6 and TNF-α production, and neutrophil infiltration	Kurashima et al. (2012)	*In vivo* animal studies and *in vitro* human studies (colon tissue from healthy patients and patients with UC and CD); colitis model using TNBS or DSS, involving anti-P2X7R mAb
Mediates activation of NLRP3 inflammasome		Hudson et al. (2019)	*In vivo* animal studies and *in vitro* human studies (using human acute monocytic leukemia cell line); involving NLRP3 and P2X receptor KO mice and P2X agonists
		Quan et al. (2018)	*In vitro* human studies involving human fetal small intestinal epithelial cells (FHs 74 Int cells); infection model with *T. gondii* involving inhibition of P2X7R
		Higashimori et al. (2016)	*In vivo* animal studies; non-steroidal anti-inflammatory drugs (NSAIDs)-induced enteropathy model involving pharmacological inhibition of P2X7R
		Chen et al. (2015)	*In vitro* animal studies involving murine macrophage-like lymphoma cell line (P388D1)
		Saber et al. (2021)	*In vivo* animal studies; colitis model using DSS involving pharmacological inhibition of P2X7R and NLRP3
		Guan et al. (2021)	*In vivo* and *in vitro* animal studies; infection model using *T. spiralis* involving pharmacological inhibition of P2X7R
Mediates activation of caspase-1 inflammasome		Liu et al. (2018)	*In vivo* and *in vitro* animal studies; infection model using *Clostridium difficile*, involving pharmacological inhibition of P2X7R and P2X7R small interfering ribonucleic acid (siRNA)
Mediating inflammatory responses to *T. gondii* infection		Miller et al. (2015)	*In vivo* animal studies; infection model using *T. gondii* involving P2X7R KO mice
Downregulates cell surface expression of glucose transporter 2 (GLUT2) expression		Bourzac et al. (2013)	*In vitro* animal studies and human cell culture; involving rat cell line IEC-6, human colon carcinoma cell line Caco-2 and human embryonic kidney cell line HEK293T; involves pharmacological inhibition of P2X7R
		Arguin et al. (2017)	*In vivo* animal studies; involving P2X7R KO mice
Modulates intestinal glucose absorption and transport into bloodstream		Arguin et al. (2017)	*In vivo* animal studies; involving P2X7R KO mice
Induces macrophages to release CD14		Alarcón-Vila et al. (2020)	*In vivo* and *in vitro* animal studies; sepsis model involving P2X7R KO mice and pharmacological inhibition of P2X7R
Involved in sepsis-induced intestinal damage		Wu et al. (2017)	*In vivo* animal studies; sepsis model involving pharmacological inhibition of P2X7R and P2X7R agonist
		Zhang et al. (2018)	*In vivo* and *in vitro* animal studies (rat intestinal epithelial cell lines IEC-6 and CRL-159) in LPS stimulation, involving pharmacological inhibition of P2X7R
Involved in metabolic regulation		Arguin et al. (2017)	*In vivo* animal studies, involving P2X7R KO mice
		Perruzza et al. (2017)	*In vivo* animal studies, microbiota transplantation involving P2X7R KO mice
		Perruzza et al. (2019)	*In vivo* animal studies, involving P2X7R KO mice
Involved in neuronal changes induced by ischemia		Palombit et al. (2019)	*In vivo* animal studies; ISR model involving pharmacological inhibition of P2X7R
Involved in alcohol-induced steatohepatitis and intestinal injury		Su et al. (2019)	*In vivo* animal studies; chronic plus binge alcohol feeding model involving pharmacological inhibition of P2X7R
Inhibitory control of colonic motility		Antonioli et al. (2014)	*In vivo* animal studies; colitis model by DNBS, involving P2X7R pharmacological inhibition and P2X7R agonist.
Anti-tumor immune response		Adinolfi et al. (2015)	*In vivo* and *in vitro* animal studies (B16 melanoma and CT26 colon carcinoma cells); tumor model involving P2X7R KO mice
Promotes proliferation of colorectal cancer (CRC) cells		Zhang et al. (2021)	*In vivo* animal studies and *in vitro* human studies on CRC cell lines (SW620 and HCT116); involving pharmacological inhibition of P2X7R and P2X7R siRNA.

## Discussion

In the GI system, it is well established that ATP released from neuronal and non-neuronal cells involves purinergic mechanosensory transduction that modulates enteric reflex activities, ion transport, tissue blood flow and pain transmission (Burnstock, 2014). The physiological role of P2X7R in the GI tract is unclear, but its unique feature of forming non-selective large pores on cell membranes in response to cell damage induced sustained increase of extracellular ATP trigger a cascade of events associated with inflammation, leading to ATP-induced recruitment and activation of lymphocytes, macrophages, and dendritic cells to the lesion sites, as well as secretion and maturation of proinflammatory cytokines (North, 2002; Trautmann, 2009). Thus, ATP and P2X7R serve as a key “danger” signal in inflammatory processes. Although P2X7R has become a new target for some chronic inflammatory disorders, to our knowledge, the only clinical trial for P2X7R antagonism was a Phase 2a randomised controlled trial using AZD9056 to treat Crohn’s disease. Even though AZD9056 improved Crohn’s Disease Activity Index scores significantly in terms of pain alleviation and patient well-being, the trial was halted due to insignificant changes in inflammatory biomarkers (Eser et al., 2015).

In the GI system, P2X7R expressed in different cell types has various actions under homeostatic and pathological conditions studied under different experimental models, e.g., colitis model (Gulbransen et al., 2012; Kurashima et al., 2012; Antonioli et al., 2014; Marques et al., 2014; Neves et al., 2014; Hofman et al., 2015; Wan et al., 2016; Figliuolo et al., 2017; Hashimoto-Hill et al., 2017; Ohbori et al., 2017; Diezmos et al., 2018; Souza et al., 2020; Saber et al., 2021), sepsis model *via* caecal ligation and puncture (CLP) (Wu et al., 2017; Alarcón-Vila et al., 2020), infection models (Keating et al., 2011; Huang et al., 2017; Shimokawa et al., 2017; Liu et al., 2018; Quan et al., 2018; Stark et al., 2018) in both animal and human studies. There were also a few studies (Bourzac et al., 2013; Proietti et al., 2014; Arguin et al., 2017; Perruzza et al., 2017; Perruzza et al., 2019; Proietti et al., 2019).

### GI Inflammation

There is strong supporting evidence that P2X7R plays a key role in GI inflammation by upregulating the production of pro-inflammatory mediators.

Eleven experimental colitis models were undertaken in rats and/or mice (Gulbransen et al., 2012; Antonioli et al., 2014; Marques et al., 2014; Hofman et al., 2015; Wan et al., 2016; Figliuolo et al., 2017; Hashimoto-Hill et al., 2017; Ohbori et al., 2017; Delvalle et al., 2018; Souza et al., 2020; Saber et al., 2021), one study involved human colonic tissue culture (Diezmos et al., 2018), and two studies involved both mouse models and colonic tissues from IBD patients (Gulbransen et al., 2012; Kurashima et al., 2012; Neves et al., 2014). To induce colitis, two studies used TNBS–25 mg in 1 ml of solution (Marques et al., 2014), 30 mg/kg in 600 μl of 30% ethanol (Souza et al., 2020). Two studies used TNBS and DSS–100 μl of 2.5% TNBS in 50% ethanol and 3.5% or 2.5% DSS in drinking water *ad libitum* (Kurashima et al., 2012); 25 mg of TNBS in 0.1 ml solution and 5% DSS in drinking water *ad libitum* (Neves et al., 2014). One study used 4% DSS in drinking water *ad libitum* (Saber et al., 2021), one study used 5% DSS in drinking water *ad libitum* and intraperitoneal (i.p.) azoxymethane (Hofman et al., 2015). One study used various concentrations of TNBS and oxazolone (Figliuolo et al., 2017), two studies used DNBS–5 mg in 0.1 ml of 50% ethanol (Delvalle et al., 2018); 30 mg in 0.25 ml of 50% ethanol (Antonioli et al., 2014). One study used DNBS (5 mg in 0.1 ml of solution), oxazolone (5 mg in 0.1 ml of solution) and DSS (3% in drinking water *ad libitum*) (Gulbransen et al., 2012). Two studies used DSS–3% (Ohbori et al., 2017) and 4% (Wan et al., 2016) in drinking water *ad libitum*. One study used pro-inflammatory cytokines (TNF-α and IL-1β, 10 ng/ml each) (Diezmos et al., 2018) and one study used i.p. administration of naïve CD4^+^ T cells (5 × 10^5^ cells/mouse) (Hashimoto-Hill et al., 2017). Twelve studies involved P2X7R antagonists (Gulbransen et al., 2012; Antonioli et al., 2014; Marques et al., 2014; Neves et al., 2014; Hofman et al., 2015; Wan et al., 2016; Figliuolo et al., 2017; Ohbori et al., 2017; Delvalle et al., 2018; Diezmos et al., 2018; Souza et al., 2020; Saber et al., 2021), four studies involved P2X7R KO mice (Neves et al., 2014; Hofman et al., 2015; Figliuolo et al., 2017; Hashimoto-Hill et al., 2017), two studies used anti-P2X7R antibodies (Kurashima et al., 2012; Neves et al., 2014), and one study used reconstituted P2X7R deficient mast cells (Kurashima et al., 2012).

P2X7R has been shown to play vital roles in intestinal inflammation in IBD and mediating colitis-induced tissue damage. P2X7R upregulated the production of pro-inflammatory cytokines (Kurashima et al., 2012; Marques et al., 2014; Neves et al., 2014; Wan et al., 2016) and mediated immune cell infiltration (Kurashima et al., 2012; Marques et al., 2014; Figliuolo et al., 2017; Ohbori et al., 2017), which propagated inflammation and contributed to microscopic and macroscopic characteristics of colitis. Kurashima et al. (2012) showed *in vitro* that P2X7R-mediated activation of mast cells played key roles in initiating and exacerbating intestinal inflammation in colitis by promoting the release of TNF-α, IL-6, leukotrienes and chemokines, and neutrophil infiltration into the colon. The work by Figliuolo et al. (2017) also provided evidence that P2X7R downregulated anti-inflammatory cytokines IL-10 and TGF-β-1. Marques et al. (2014) and Neves et al. (2014) found that P2X7R was involved in intestinal epithelial cell apoptosis, while Delvalle et al. (2018) showed that P2X7R activation was involved in the neurodegeneration of enteric neurons during inflammation. Gulbransen et al. (2012) also determined that P2X7R had a role in mediating enteric neuronal death, suggesting the involvement of P2X7R in colonic motor dysfunction in the setting of inflammation. Increased P2X7R expression has been reported in murine models of colitis (Kurashima et al., 2013; Marques et al., 2014; da Silva et al., 2015). In concordance with the study by Gulbransen et al. (2012) and Antonioli et al. (2014) reported that due to increased expression during colitis, P2X7R took a more prominent role in modulating colonic motility and was involved in the tonic inhibitory control on excitatory cholinergic motility. Figliuolo et al. (2017) found that P2X7R triggered the death and retention of Treg cells in the mesenteric lymph nodes (MLN) that suppressed activation and effector functions of other immune cells, thereby preventing immune system tolerance in the GI system.

There were many different variables across these papers, including different chemicals to induce colitis, different methods to inhibit P2X7R, different rat or mouse types, making it difficult to compare the significance of their findings in relation to one another. The study by Figliuolo et al. (2017) is the only paper to report a decrease in anti-inflammatory cytokines due to P2X7R activation in experimental colitis; more studies to determine the reproducibility of their results would further substantiate this action of P2X7R. The chemicals used to induce colitis is arguably the most important distinctive factor. TNBS-induced colitis is similar to human CD and results in mononuclear inflammatory infiltrate in the colon wall (Strober et al., 2002); DSS-induced colitis leads to disruptions in the intestinal epithelial layer, increasing the risk of exposure to micro-organisms in the lumen which would result in activation of mucosal dendritic cells and macrophages and subsequent up-regulation of pro-inflammatory cytokines (Kitajima et al., 1999; Strober et al., 2002). Regardless, both TNBS and DSS induce extensive colitis with intestinal inflammation, diarrhoea and significant weight loss (Neves et al., 2014). Despite the variability, their findings support that P2X7R is involved in the aberrant inflammation, intestinal damage, and subsequent consequences on GI dysmotility in IBD. This also strongly suggests that P2X7R antagonism can have therapeutic benefits.

In the studies involving P2X7R antagonists, Marques et al. (2014) showed that prophylactic systemic administration of P2X7R antagonist, e.g., Brilliant Blue G (BBG, 40 mg/kg) or A740003 (16 mg/kg) 24 h prior to TNBS injection, was highly efficacious in attenuating colitis-induced tissue damage, apoptosis and inflammatory responses. Souza et al. (2020) described improvements in clinical signs of colitis in the BBG-treated group (50 mg/kg administered 1 h following TNBS injection) (Souza et al., 2020). Ohbori et al. (2017) also found that per oral (p.o.) administration of BBG (250 mg/kg; daily from a day before DSS injection) was effective in alleviating the progression of DSS-induced colitis by significantly decreasing the rate of increase in disease activity index (DAI), preventing colon shortening, diminishing microscopic changes due to DSS and reducing mast cell accumulation in the colon. Diezmos et al. (2018) found that the P2X7R antagonist A438079 (100 μM) decreased cytokine-induced crypt damage but was less effective in restoring levels of tight junction protein zonula occludens-1 (ZO-1) and maintaining epithelial cell integrity. Saber et al. (2021) showed that BBG treatment (50 mg/kg, p.o.) significantly increased mRNA levels of tight junction proteins occludin (Ocln) and ZO-1 compared to untreated groups; additionally, combined treatment using BBG and an NLRP3 inflammasome inhibitor OLT1177 (20 mg/kg, p.o.) significantly increased mRNA expression of ZO-1 and Ocln to similar levels of control mice without DSS-induced colitis. Wan et al. (2016) showed that A438079 attenuated chemically-induced colitis by decreasing levels of pro-inflammatory cytokines, improving stool consistency, presence of blood in feces and weight loss, and reducing microscopic tissue damage. Gulbransen et al. (2012) demonstrated that prophylactic inhibition of P2X7R by oxidised-ATP (oATP, 7.5 mg/kg i.p.) reduced inflammation-induced neuronal loss but did not inhibit inflammation-induced macroscopic tissue damage or weight loss. Delvalle et al. (2018) showed that 10 μmol/L of A74003 reduced neuronal loss by 77% when challenged by neurokinin which mediates inflammation in the mucosa *in situ*. Different P2X7R inhibitors of various concentrations were used in different chemically-induced colitis experiments across these papers. Overall, inhibition of P2X7R rescued colitis-induced intestinal damage, disruption of the epithelial layer, reduced expression of inflammatory cytokine expression and decreased clinical signs of colitis such as diarrhoea or weight loss.

Kurashima et al. (2012) showed that following TNBS-induction, mice reconstituted with P2X7R^-/-^ mast cells had reduced inflammatory responses, including decreased mast cell activation and neutrophil infiltration in the colon compared to mice with P2X7R^+/+^ mast cells. This implies that P2X7R-mediated activation of mast cells is involved in exacerbated inflammation seen in TNBS-induced colitis (Kurashima et al., 2012). Similar to mice with P2X7R^-/-^ mast cells, there was no increase in inflammatory cytokines, leukotrienes and neutrophil infiltration *in vitro* in P2X7R blockade using 1F11 mAb (anti-P2X7R mAb) (Kurashima et al., 2012). Figliuolo et al. (2017) showed that P2X7R KO reduced immune cell infiltration, prevented Treg cell death and overall protected against the development of colitis; administration of the P2X7R antagonist A740003 in WT mice also protected against chemically-induced colitis. Neves et al. (2014) found that P2X7R deficiency protected against intestinal damage in chemically-induced colitis with decreased cellular infiltration and microscopic damage, and maintained body weight in P2X7R KO mice compared to WT littermates. The effects of P2X7R KO in chemically-induced colitis are similar to that of P2X7R blockade using antagonists or anti-P2X7R antibodies.

Nevertheless, Hofman et al. (2015) found a link between P2X7R antagonism (using A438079) and colitis-associated cancer. They found that A438079-treated mice had greater numbers of macroscopic polyps and significantly larger tumors than the untreated mice. This was possibly due to increased TGF-β1 secretion in P2X7R blockade, which reportedly increases colonic epithelial cell proliferation (Yan et al., 2002), thus contributing to the development of colonic tumors and malignancy (Cesaro et al., 2010). This was the only paper to study chemically-induced colitis with colitis-associated cancer, which was induced using azoymethane over a duration of 2 months. This was significantly longer than other studies which lasted from 1 (Souza et al., 2020) to 11 days (Ohbori et al., 2017), allowing Hofman et al. (2015) to examine the longer term effects of P2X7R blockade in chemically-induced colitis. The long-term implications seen here are important to consider with the growing interest in using P2X7R blockade to treat IBD.

Other papers on P2X7R in the GI system that do not involve chemically-induced colitis still highlight important roles of P2X7R when activated. de Campos et al. (2012) found that P2X7R, when activated by ATP, upregulated pro-inflammatory mediator IL-1β and resulted in increased permeability in lymphocytes and macrophages in the MLN and colonic epithelial cells. Several papers highlight how P2X7R activation contributes to the loss of IEC barrier integrity through increased permeability of IECs (de Campos et al., 2012), loss of tight junction proteins (Diezmos et al., 2018; Su et al., 2019) and apoptosis of IECs (Souza et al., 2012; Marques et al., 2014). This intestinal barrier dysfunction is important in the pathogenesis of IBD (Coskun, 2014). Interestingly, Hashimoto-Hill et al. (2017) showed that P2X7R activated by nicotinamide adenine dinucleotide (NAD) suppressed colitis, decreased weight loss and inflammation-related colon shortening. They found that P2X7R controls the Th1 and Th17 cell populations by inducing apoptosis, and activation of P2X7R by NAD depleted inflammatory T cells in the small intestines (Hashimoto-Hill et al., 2017). These findings differ from the majority of the papers that show an inflammatory role of P2X7R. This could be attributed to significantly different methods employed in this part of their work where they compared Rag1 deficient mice that do not have mature B or T cells (Mombaerts et al., 1992) to their wild-type (WT) counterparts instead of comparing the severity of inflammation and colitis in P2X7R KO mice versus WT mice in examining the role of NAD-activated P2X7R (Hashimoto-Hill et al., 2017).

Taken together, P2X7R appears to be highly involved in the development of IBD, which makes P2X7R blockade of particular interest for its therapeutic management. However, the areas of concern regarding P2X7R antagonism are the exacerbated inflammation (Hashimoto-Hill et al., 2017) and association with the development of tumors and cancers of the colon (Hofman et al., 2015). Additionally, the findings by Saber et al. (2021) highlights the potential of combined treatment of a P2X7R blocker with other antagonists of molecules involved in the inflammatory process since the combination of BBG and OLT1177, an NLRP3 inhibitor, was more effective at protecting against DSS-induced colitis than their individual administration. Further research on the actions of P2X7R and reproducing the results by Hofman et al. (2015) and Hashimoto-Hill et al. (2017) to confirm their conclusions is essential in our understanding of these differencing actions of P2X7R and the long-term effects of P2X7R antagonism.

### Sepsis

One of the severe complications of IBD is sepsis. Two sepsis models using rats and mice were reported (Wu et al., 2017; Alarcón-Vila et al., 2020), one of which also involved human blood samples (Alarcón-Vila et al., 2020). For their *in vivo* animal studies, the CLP procedure was done by both papers. Wu et al. (2017) ligated the caecum 1.5 cm from the cecal tip, while Alarcón-Vila et al. (2020) ligated 2/3 of the mouse caecum. The greater the distance of the ligature from the cecal tip, the more severe the sepsis, with ligation at 75% of the caecum resulting in high-grade sepsis and 100% mortality (Rittirsch et al., 2009). Thus, Alarcón-Vila et al. (2020) induced a higher grade of sepsis than Wu et al. (2017). The study by Wu et al. (2017) involved the i.p. administration of P2X7R agonist (BzATP) and antagonist (A740003) 24 h after CLP. Alarcón-Vila et al. (2020) included both P2X7R KO mice and P2X7R antagonist (A438079).

Wu et al. (2017) found that P2X7R activation by BzATP resulted in increased pro-inflammatory cytokines, higher histological inflammatory scores, increased intestinal permeability, greater IEC apoptosis and greater bacterial translocation to the MLN, caudal lymph nodes, blood and peritoneal fluid compared to the sham group. The tight junctions in the BzATP-treated group were not visible, paracellular spaces were greater and there were reduced intestinal tight junction proteins (Wu et al., 2017). These results demonstrated that P2X7R promoted inflammation and played a key role in sepsis-induced intestinal damage (Wu et al., 2017). Conversely, Alarcón-Vila et al. (2020) provided evidence that P2X7R is involved in preventing bacterial dissemination during sepsis and decreased mortality. ATP-activated P2X7R on LPS-primed macrophages induced the release of extracellular CD14; evidence suggests that CD14 was essential for decreasing bacterial dissemination and increasing survival rates (Alarcón-Vila et al., 2020).

Wu et al. (2017) showed that in CLP-induced sepsis animals, A740003 treatment significantly decreased IL-6 and TNF-α levels compared to non-treated animals. A740003 also protected against sepsis-induced intestinal barrier disruption; the protective effects of P2X7R antagonism are partially through inhibiting the activation of M1 macrophages *via* the ERK and NF-κB signaling pathways (Wu et al., 2017). In contrast, Alarcón-Vila et al. (2020) reported a higher level of cytokine production during sepsis in P2X7R KO animals than WT littermates. In addition, P2X7R KO mice and WT mice treated with A437089 had more severe signs of liver, spleen and lung damage than WT littermates (Alarcón-Vila et al., 2020). In the course of sepsis and inflammatory responses, macrophages undergo “adaptation” and develop immunosuppressive features, including reduced levels of pro-inflammatory cytokines (López-Collazo et al., 2014). This could be a contributing factor to the outlier result seen in the study by Alarcón-Vila et al. (2020), where high-grade sepsis was induced and P2X7R appeared to downregulate cytokine production *via* the increase in extracellular CD14.

The study by Zhang et al. (2018) investigated sepsis-induced intestinal injury through LPS challenge using LPS and dioleoyl-3-trimethylammonium propane *in vitro* and i.p. 10 mg/kg of LPS from *Escherichia coli* O111:B4 *in vivo*. P2X7R was found to be involved in intestinal enterocyte pyroptosis, microscopic intestinal damage, injury to intestinal epithelial lining and villus length, thus contributing to sepsis-induced disruption of intestinal mucosal barrier and epithelial structure (Zhang et al., 2018). These effects were improved in the group treated with P2X7R antagonist (i.p. 80 mg/kg A438079), which also significantly improved survival time and mortality with LPS challenge (Zhang et al., 2018).

The effect of P2X7R activation appears to be conflicting, although this could be due to the difference in the severity of sepsis induced. Overall, further research is needed to investigate the discrepancies in findings if P2X7R is to be considered a therapeutic target in the setting of sepsis.

### GI Infection

There were eleven GI infection models undertaken in mice (Heiss et al., 2008; Keating et al., 2011; Coquenlorge et al., 2014; Miller et al., 2015; Huang et al., 2017; Shimokawa et al., 2017; Liu et al., 2018; Stark et al., 2018; Guan et al., 2021; Kataoka et al., 2021), human fetal small intestinal epithelial cells (FHs 74 Int cells) (Quan et al., 2018), human liver and spleen cell samples (Stark et al., 2018), human longitudinal muscle-myenteric plexus (Coquenlorge et al., 2014).

Infective agents used included *T. gondii* (Miller et al., 2015; Huang et al., 2017; Quan et al., 2018), *T. spiralis* (Keating et al., 2011; Huang et al., 2017; Guan et al., 2021), *Heligmosomoides polygyrus* (Hp) (Shimokawa et al., 2017), lymphocytic choriomeningitis (LCMV) (Stark et al., 2018), *C. difficile* (Liu et al., 2018), *C. koseri* (Kataoka et al., 2021), *L. monocytogenes* (Heiss et al., 2008), and LPS from *E. coli* and *Salmonella typhosa* (Coquenlorge et al., 2014). Modes of administration included orally (Heiss et al., 2008; Keating et al., 2011; Miller et al., 2015; Huang et al., 2017; Shimokawa et al., 2017; Stark et al., 2018) or intraperitoneally (Stark et al., 2018) for *in vivo* murine models. These studies involved P2X7R KO mice (Heiss et al., 2008; Keating et al., 2011; Miller et al., 2015; Huang et al., 2017; Stark et al., 2018), pharmacological inhibition of P2X7R (Coquenlorge et al., 2014; Huang et al., 2017; Shimokawa et al., 2017; Liu et al., 2018; Stark et al., 2018; Guan et al., 2021; Kataoka et al., 2021), P2X7R siRNA (Liu et al., 2018; Quan et al., 2018), ARTC2.2-blocking nanobody s+16a (Stark et al., 2018) and anti-P2X7R antibodies (Heiss et al., 2008).

P2X7R has been shown to promote and enhance immune responses in infections and P2X7R blockade or deficiency would have detrimental effects on infection control. There is evidence that P2X7R is essential for the secretion of IL-1β in modulating immune responses in infection (Keating et al., 2011; Liu et al., 2018; Quan et al., 2018). P2X7R was involved in the activation of NLRP3 inflammasome in small intestinal epithelial cells during *T. gondii* infection (Quan et al., 2018) and in macrophages in *T. spiralis* infection (Guan et al., 2021); activation of NLRP3 inflammasome increased IL-1β secretion, which was important in limiting the proliferation of infective agents (Quan et al., 2018; Guan et al., 2021). Inhibiting P2X7R significantly reduced NLRP3 inflammasome activation, in turn decreasing IL-1β and increasing intracellular parasite burden (Quan et al., 2018; Guan et al., 2021). Similarly, Liu et al. (2018) showed that P2X7R was essential for the secretion of IL-1β *via* activating caspase-1 inflammasome. Although they did not study the subsequent effects of *C. difficile* infection in the setting of P2X7R deficiency, they did find that inhibition of caspase-1 inflammasome activation led to more severe disease progression with greater weight loss, shorter colon length and increased bacterial load in feces and caecum (Liu et al., 2018). Inhibition of P2X7R would likely have similar results due to its role in caspase-1 inflammasome activation.

Huang et al. (2017) determined a key role of P2X7R in immune responses to *T. gondii* and *T. spiralis* infection that decreased parasitic burden. They found that P2X7R was involved in dendritic cell infiltration to the site of infection, promoting TNF-α and IL-6 production in IECs, and promoting specific T cell responses during infection (Huang et al., 2017). P2X7R KO mice had impaired chemotaxis, reduced dendritic cell recruitment, reduced specific T cell responses and an overall increased parasitic burden (Huang et al., 2017). Guan et al. (2021) also found that P2X7R KO mice had increased worm burden and intestinal damage in *T. spiralis* infection. Shimokawa et al. (2017) found that ATP-activated P2X7R on mast cells in IEC damage induced by Hp infection increased IL-33 production. IL-33 is an important damage-associated molecular pattern (DAMP) and is essential for the protection against Hp (Shimokawa et al., 2017). Inhibition of P2X7R using BBG increased the susceptibility of mice to Hp infection, denoted by an increase in Hp eggs in feces (Shimokawa et al., 2017).

Miller et al. (2015) found that *T. gondii*-infected P2X7R KO mice experienced a more severe infection with greater swelling, angiogenesis, presence of pus, microscopic intestinal damage and disruption of intestinal epithelial structure than infected WT littermates. Interestingly, this was in association with increased secretion of pro-inflammatory cytokines IL-12, interferon-γ, TNF, IL-1β and IL-6 despite impaired NF-κB pathway activation in P2X7R KO mice (Miller et al., 2015). Although Miller et al. (2015) found that P2X7R deficiency led to diminished immune responses in infection and poorer outcomes, their findings also demonstrated a possible anti-inflammatory role of P2X7R. As the paper by Miller et al. (2015) measured the inflammatory response in an infection setting, it could contribute in its conflicting results to the other colitis model papers, which purely examined inflammation in chemically-induced colitis. However, this does not account for the differing results from other infection models showing P2X7R-mediated inflammatory responses in clearing infections (Keating et al., 2011; Liu et al., 2018; Quan et al., 2018).

It is well known that *T. gondii* is an intracellular protozoan parasite that can evade host immune responses by inhibiting apoptosis and inhibiting the expression of IFN-γ and NF-κB pathways, leading to diminished production of pro-inflammatory cytokines (Lima and Lodoen, 2019). The diminished susceptibility to *T. gondii* induced ileitis in wild type mice compared to P2X7R KO mice observed by Miller et al. (2015) appears irrelevant to the immune evasion ability of T. gondii, since both groups showed similar efficacy in controlling the parasite. Instead, susceptibility to ileitis in P2X7R KO mice is associated with elevated production of pro-inflammatory cytokines in the ileum. Thus, Miller et al. (2015) concluded that there could be a possible anti-inflammatory role of P2X7R (Miller et al., 2015).

Conversely, there is evidence that P2X7R contributes to diminishing immune responses, and P2X7R blockade could be beneficial in promoting immunity in infections. Stark et al. (2018) found that in the homeostatic control of T_RM,_ T cell receptor mediated activation of T_RM_ reduced its expression of P2X7R, thereby decreasing T_RM_ cell death induced by activation of P2X7R. However, IL-12 upregulated P2X7R expression during infection-induced inflammation, thus sensitizing T_RM_ to apoptosis, while T_RM_ depletion prevented further immune activation (Stark et al., 2018). Similarly, *in vitro* experiments showed that tissue damage in the liver depletes T_RM_ *via* P2X7R activation (Stark et al., 2018). Hence, targeting P2X7R and modulating its expression on T_RM_ during infections could ensure the maintenance of specific T_RM_ cells and enhance immune responses. Hashimoto-Hill et al. (2017) found that P2X7R KO mice showed increased Th1 and Th17 cell populations and experienced more severe inflammatory responses to infection by *C. rodentium*. However, whether the more severe inflammatory response was beneficial in clearing infection or detrimental and induced significant intestinal tissue damage when compared with WT littermates was not explored. The study by Coquenlorge et al. (2014) demonstrated that LPS-induced TNF-α production in the enteric nervous system was inhibited by ATP-activated P2X7R. Their *in vitro* data suggests a possible role of the enteric nervous system in gastrointestinal inflammatory responses during infectious and inflammatory challenges.

While most papers agree that P2X7R is essential in modulating immune responses in infections and controlling or eliminating infection (Huang et al., 2017; Shimokawa et al., 2017; Quan et al., 2018; Guan et al., 2021), there are still papers with conflicting results, showing that P2X7R may not be important in controlling infection (Heiss et al., 2008) or that P2X7R-activation suppressed immune activation (Stark et al., 2018). Once again, these infection-model papers are extremely variable with different infective agents and different cell types examined. This highlights the need for further research to establish a clear role of P2X7R in mediating immune responses and eliminating infections in animal and human models.

### Other Roles of P2X7R in the GI System

Apart from its role in the inflammatory process and modulating immune responses, P2X7R has also been shown to have a role in GI malignancies (Adinolfi et al., 2015; Hofman et al., 2015; Zhang et al., 2021), immune modulation (Proietti et al., 2014; Perruzza et al., 2017; Liu and Kim, 2019; Perruzza et al., 2019; Proietti et al., 2019), metabolic regulation (Bourzac et al., 2013; Arguin et al., 2017; Perruzza et al., 2017; Perruzza et al., 2019), ISR injury (Palombit et al., 2019) and intestinal damage due to long-term morphine use (Bhave et al., 2017), chronic binge alcohol feeding (Su et al., 2019) and NSAID use (Higashimori et al., 2016).

Similar to the findings by Hofman et al. (2015) that showed enhanced colitis-associated cancer tumor development in P2X7R blockade, Adinolfi et al. (2015) found that P2X7R is essential for anti-tumor immune responses to restrict tumor growth and metastasis. P2X7R KO mice reduced activation of dendritic cells in response to tumor cells, resulting in diminished chemotaxis and inflammatory infiltration in tumors and surrounding tissues of P2X7R KO mice compared to WT littermates (Adinolfi et al., 2015). P2X7R blockade led to decreased immune responses; this was associated with larger tumor sizes and increased rate of metastasis in B16 mouse melanoma and CT26 mouse colon carcinoma cells in P2X7R KO mice compared to WT littermates (Adinolfi et al., 2015). Conversely, Zhang et al. (2021) found *in vivo* and *in vitro* that P2X7R was implicated in the proliferation and metastasis of CRC cancer. Pharmacological blockade using A438079 (10 μM) and AZD9056 (10 μM) or transfecting CRC cell lines with P2X7R siRNA reduced CRC proliferation by inhibiting P13/Akt/GSK-3-β/β-catenin pathway which is an important signaling pathway in tumor development (Zhang et al., 2021). In concordance with the results by Zhang et al. (2021), studies have found a correlation between higher P2X7R expression and worse prognosis in CRC (Zhang et al., 2019), hepatocellular carcinoma, adenocarcinoma and ampullary carcinoma (Asif et al., 2019). These papers studying the role of P2X7R in GI malignancies involve different cancer cell lines for various durations [21 days (Adinolfi et al., 2015), 1 month (Zhang et al., 2021) and 2 months (Hofman et al., 2015)], making it difficult to weigh the significance of their findings. However, the conflicting results necessitate further studies on the therapeutic potential of P2X7R blockade in dampening tumor progression and the effects of long-term P2X7R blockade in the development of colitis-associated cancer.

P2X7R has been shown to play a role in controlling the microecology of GI bacteria (Proietti et al., 2014; Perruzza et al., 2017; Perruzza et al., 2019; Proietti et al., 2019). ATP from gut bacteria activated P2X7R which limited Tfh cell populations (Proietti et al., 2014; Perruzza et al., 2019; Proietti et al., 2019). This in turn modulated secretory immunoglobulin A (SIgA) activity to build tolerance to commensal bacteria (Perruzza et al., 2019; Proietti et al., 2019). Transient inactivation of P2X7R could be used to enhance SIgA responses and reduce intestinal inflammation in oral vaccinations (Proietti et al., 2019). However, long-term P2X7R deficiency has detrimental effects on immune responses—Proietti et al. (2014) found that P2X7R-deficient Tfh cells led to high-affinity IgA responses that bind to and deplete commensal gut mucosal bacteria. This led to increased susceptibility to polymicrobial sepsis induced by CLP in P2X7R KO mice due to decreased B1 cell stimulation and serum immunoglobulin M (IgM) concentrations (Proietti et al., 2014). Additionally, P2X7R deficiency alters the microecology of gut microbiota (Proietti et al., 2014; Perruzza et al., 2017; Perruzza et al., 2019; Proietti et al., 2019); this was associated with glucose and lipid dysregulation (Perruzza et al., 2017; Perruzza et al., 2019). Perruzza et al. (2019) demonstrated that P2X7R KO mice had altered gut microecology, higher white adipose tissue, body weight, blood glucose, perigonadal fat and decreased glucose clearance than WT littermates. These reported roles of P2X7R on SIgA, composition of gut microbiota and subsequent effects on glucose and lipid homeostasis pose a challenge to using P2X7R antagonists to treat IBD or other GI pathologies. Further studies should examine changes in metabolism and susceptibility to infections in experimental colitis models.

Apart from its role in glucose homeostasis *via* affecting the composition of gut microbiota (Perruzza et al., 2019), it has been reported that P2X7R activation modulates glucose absorption by downregulating cell surface GLUT2 expression in enterocytes (Bourzac et al., 2013). This resulted in decreased glucose absorption into the blood (Bourzac et al., 2013). The findings from Arguin et al. (2017), built upon the paper by Bourzac et al. (2013), showed that P2X7R KO mice increased apical and basolateral expression of GLUT2 on enterocytes. This was accompanied by increased weight gain, elevated blood glucose levels, triglyceride concentration, cholesterol concentrations and serum insulin levels (Arguin et al., 2017). P2X7R KO mice also developed hepatic steatosis with increased lipid accumulation in hepatocytes but decreased expressions in genes associated with liver lipid metabolism (Arguin et al., 2017). With increasing interest in the therapeutic uses of P2X7R antagonists, it is imperative to consider effects on metabolism in light of evidence showing P2X7R’s role in metabolic regulation.

In a chronic binge alcohol feeding model in mice by Su et al. (2019), 5% (vol/vol) ethanol was used for 10 days and controls were pair-fed. I.p. administration of BBG (25, 50 and 100 mg/kg) or A438079 (100 mg/kg) daily from day 4–10 was used to block P2X7R (Su et al., 2019). They showed that P2X7R was involved in alcohol feeding-induced steatohepatitis and intestinal injury. This was associated with the upregulation of inflammatory cytokines (TNF-α, IL-1β and IL-6) in intestinal and liver tissues, and decreased expression of tight junction proteins ZO-1 and claudin-1 in intestinal tissues (Su et al., 2019). BBG (100 mg/kg) or A438079 reduced alcohol-induced steatohepatitis and intestinal injury by reducing inflammatory cytokine production, reducing neutrophil infiltration in the liver and preventing intestinal barrier disruption. Gut microbiota composition was altered in chronic plus binge alcohol feeding; these alterations were recovered with P2X7R blockage treatment (Su et al., 2019). The findings here are in line with the aforementioned studies that support an inflammatory role for P2X7R and its control over gut micro-ecology. P2X7R antagonism has potential therapeutic benefits for a wide variety of GI pathologies involving inflammation and intestinal damage; however, the adverse effects on the gut bacterial composition, metabolism, and GI malignancies should be examined thoroughly.

**FIGURE 2:**
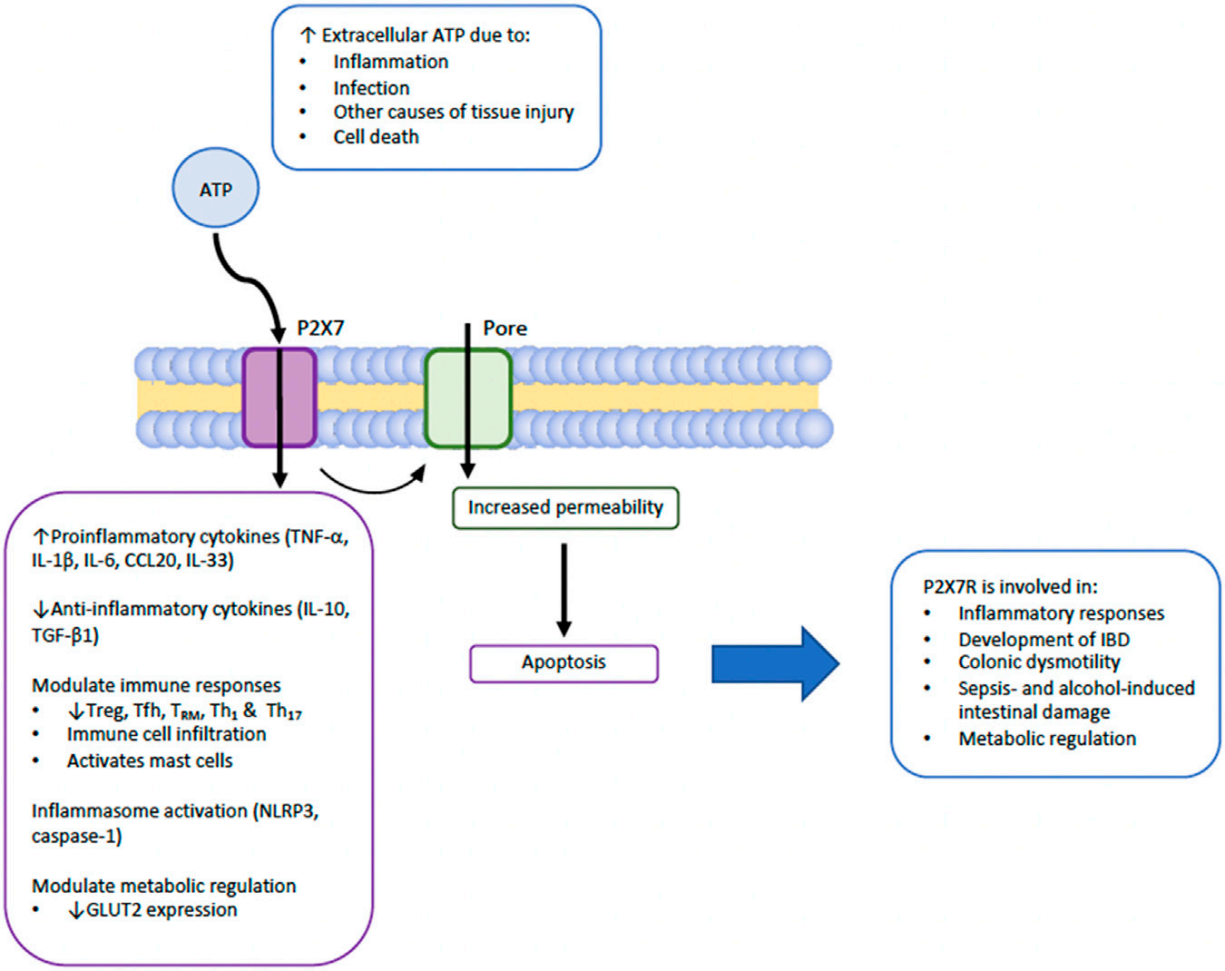
Summary of the primary actions of P2X7R in the GI system.

Taken together, as summarized in Figure 2, P2X7R plays important roles in gastrointestinal inflammation and infection through upregulating proinflammatory cytokines (TNF-α, IL-1β, IL-6, CCL20, IL-33), downregulating anti-inflammatory cytokines (IL-10, TGF-β1), modulating immune responses, inflammasome activation, and increasing permeability of the cell leading to increased apoptosis. P2X7R has also been implicated in certain GI malignancies, although whether it is positively or negatively involved in tumour progression has yet to be determined in the various cancer cell lines studied (Adinolfi et al., 2015; Hofman et al., 2015; Zhang et al., 2019; Zhang et al., 2021).

### Limitations

A limitation of this systematic review is that it focuses on homeostatic and pathological conditions of the GI system. P2X7R is highly expressed in various tissues and organs, and its role is not limited to the GI only (Burnstock, 2012). Secondly, papers that do not focus on the role of P2X7R were not included. This could have excluded a few findings, but it helped narrow down the scope of papers to analyze for a comprehensive discussion on the role of P2X7R in the GI system. Additionally, papers that did not focus on P2X7R might not provide sufficient evidence to substantiate the role of P2X7R. Finally, the majority of the experimental-colitis studies available were animal models, and a wide variety of chemicals were used to induce colitis. This made it difficult to compare the outcomes of these studies and to postulate the effectiveness of P2X7R blockade in human models.

## Conclusion

In conclusion, P2X7R is expressed in many cell types and plays important physiological and pathophysiological roles in the GI system. It can regulate immune cell populations under normal conditions, mediate immune responses when challenged by infection, inflammation or other forms of tissue injury. Differing observations on the roles of P2X7R have also been reported. For instance, although the majority of papers agree on its role in upregulating inflammation, there are reports of P2X7R involved in downregulating inflammation and suppressing colitis. P2X7R can enhance or suppress immune responses in GI infections, and suppress or promote tumor development. P2X7R antagonism has potential for therapeutic uses; however, from differing evidence on its role, P2X7R inhibition can have beneficial or detrimental effects in IBD, sepsis, infections and malignancies. The effects on gut microbiota composition and glucose and lipid metabolism are also important considerations in P2X7R blockade. For a more comprehensive understanding of P2X7R’s actions and the effects of its blockade, further research in GI and other systems is crucial, especially in light of conflicting reports on its role.

## Data Availability

The original contributions presented in the study are included in the article, further inquiries can be directed to the corresponding author.

## Glossary

ATP: adenosine triphosphate
BBG: Brilliant Blue G;
CCL20: chemokine ligand 20
CD: Crohn’s disease
CLP: caeca ligation and puncture
DAMP: danger-associated molecular pattern;
DNBS: 2,4-Dinitrobenzene sulphonic acid
DSS: dextran sulphate sodium
EMT: epithelial-mesenchymal transition
ERK: extracellular signal-regulated kinase
GI: gastrointestinal
GLUT2: glucose transporter 2
Hp: *Heligmosomoides polygyrus*
IBD: inflammatory bowel disease
IEC: intestinal epithelial cell
IL: interleukin
IgA: immunoglobulin A
IgM: immunoglobulin M
iNKT: invariant natural killer cells
i.p.: intraperitoneal
ISR: ischemia reperfusion injury
KO: knockout
LCMV: lymphocytic choriomeningitis
LPS: lipopolysaccharide
mAB: monoclonal antibody
MLN: mesenteric lymph nodes
NAD: nicotinamide adenine dinucleotide
NF-κB: nuclear factor kappa-light-chain-enhancer of activated B cells
NSAID: non-steroidal anti-inflammatory drug
oATP: oxidized ATP
Ocln: occluding
P2X7R: P2X7 receptor
p.o.: per oral
ROS: reactive oxygen species
SIgA: secretory immunoglobulin A
siRNA: small interfering ribonucleic acid
Tfh: T follicular helper
TGF: transforming growth factor
TNBS: 2,4,6-trinitrobenzenesulfonic acid
TNF: tumor necrosis factor
Treg: regulatory T cells
**T**_**RM**_: T
UC: ulcerative colitis
WT: wild-type
ZO-1: zonula occludens-1
